# Advances in translational bioinformatics facilitate revealing the landscape of complex disease mechanisms

**DOI:** 10.1186/1471-2105-15-S17-I1

**Published:** 2014-12-16

**Authors:** Jack Y Yang, A Keith Dunker, Jun S Liu, Xiang Qin, Hamid R Arabnia, William Yang, Andrzej Niemierko, Zhongxue Chen, Zuojie Luo, Liangjiang Wang, Yunlong Liu, Dong Xu, Youping Deng, Weida Tong, Mary Qu Yang

**Affiliations:** 1 Joint Bioinformatics Ph.D. Program, George W. Dougherty College of Engineering and Information Technology, University of Arkansas at Little Rock and University of Arkansas for Medical Sciences, 2801 S. University Avenue, Little Rock, Arkansas 72204, USA; 2 Division of Biostatistics and Biomathematics, Department of Radiation Oncology, Massachusetts General Hospital and Harvard Medical School, Boston, Massachusetts 02114, USA; 3 Center for Computational Biology and Bioinformatics, Indiana University School of Medicine, Indianapolis, Indiana 46202, USA; 4 Department of Statistics, Harvard University, Cambridge, Massachusetts 02138, USA; 5 Human Genome Sequencing Center, and Department of Molecular and Human Genetics, Baylor College of Medicine, Houston, Texas 77030, USA; 6 Department of Computer Science, University of Georgia, Athens, Georgia 30602, USA; 7 Department of Computer Science, George W. Donaghey College of Engineering and Information Technology, University of Arkansas at Little Rock, 2801 S. University Ave, Little Rock, Arkansas 72204, USA; 8 Department of Epidemiology and Biostatistics, Indiana University School of Public Health, 1025 E. 7th Street, PH C104, Bloomington, Indiana 47405, USA; 9 Department of Endocrinology, Guangxi Medical University First Affiliated Hospital. Nanning, Guangxi 530021, China; 10 Department of Genetics and Biochemistry, Clemson University, Clemson, South Carolina 29634, USA; 11 Department of Computer Science, University of Missouri, Columbia, Missouri 65211, USA; 12 Rush University Cancer Center, and Departments of Internal Medicine and Biochemistry, Rush University Medical Center, Chicago, Illinois 60612, USA; 13 Divisions of Bioinformatics and Biostatistics, National Center for Toxicological Research, United States Food and Drug Administration, 3900 NCTR Road, Jefferson, Arkansas 72079, USA; 14 MidSouth Bioinformatics Center, Department of Information Science, George W. Donaghey College of Engineering and Information Technology, University of Arkansas at Little Rock, 2801 S. University Avenue, Little Rock, Arkansas, 72204, USA; 15 Joint Bioinformatics Graduate Program, University of Arkansas at Little Rock and University of Arkansas for Medical Sciences, Little Rock, Arkansas, 72204, USA

## Abstract

Advances of high-throughput technologies have rapidly produced more and more data from DNAs and RNAs to proteins, especially large volumes of genome-scale data. However, connection of the genomic information to cellular functions and biological behaviours relies on the development of effective approaches at higher systems level. In particular, advances in RNA-Seq technology has helped the studies of transcriptome, RNA expressed from the genome, while systems biology on the other hand provides more comprehensive pictures, from which genes and proteins actively interact to lead to cellular behaviours and physiological phenotypes. As biological interactions mediate many biological processes that are essential for cellular function or disease development, it is important to systematically identify genomic information including genetic mutations from GWAS (genome-wide association study), differentially expressed genes, bidirectional promoters, intrinsic disordered proteins (IDP) and protein interactions to gain deep insights into the underlying mechanisms of gene regulations and networks. Furthermore, bidirectional promoters can co-regulate many biological pathways, where the roles of bidirectional promoters can be studied systematically for identifying co-regulating genes at interactive network level. Combining information from different but related studies can ultimately help revealing the landscape of molecular mechanisms underlying complex diseases such as cancer.

## Introductory review

"On the Origin of Species", which was published in 1859 authored by Charles Darwin, laid the foundation of theory of evolution. Modern evolutionary biology has been now illustrated through genetic variations among individuals. Today, studies showed that genetic alterations can cause diseases, yet the driver mutations that cause complex diseases such as cancer remain unclear. Disease-driving genomic alterations can be inferred from comprehensive studies of genomic data and gene network analysis.

It has been known that many identified mutation-based drug targets have unwanted side effects of inhibitor treatment that often cause resistance to drugs. Studies of IDP (intrinsically disordered proteins) showed that due to differences in the post-translational circuitry such as the phosphorylation networks, where phosphorylation sites are typically within IDP regions of motifs that are dynamically unconserved during evolution. Genetically altered cells such as cancerous cells often have aggregated mutations in the target kinase. Evolutionary divergence of phosphorylation and functional alterations in protein kinases are likely correlated and evolutionary conserved kinase substrate interactions in phosphoproteins are more likely mutated in cancer. Genomic mutation-induced rewiring of the signalling networks can be prone to complex diseases such as cancer. It is important to identify IDP and evolutionary conserved networks that affect diseases for the identification of underlying disease mechanisms and more effective drug targets.

Equally important are the identification of bidirectional promoters and the regulation of genes associated with mutations or dysregulation in cancer that are enriched with bidirectional promoters. Those genes such BRCA1, BRCA2, BARD1, FANCA, FANCF, FANCB, FANCD2, P53, ERBB2, and CHEK2 work together as a group sharing regulation by bidirectional promoters and were found prevalence in ETS family factors. Identification of biological roles in bidirectional promoters is essential to our understanding of the regulatory mechanisms of bidirectional promoters and how they can regulate cancer genes. While common transcription binding factors were found in these genes, co-expression networks in human cancer can often be used to infer the underlying disease mechanisms from bidirectional promoters in combination with gene expression profile.

Furthermore, advances in high throughput RNA sequencing technologies have generated large volumes of gene expression data from RNA-Seq in addition to the microarray DNA-chip data. Genomic mutations in disease-causing genes or structural variations in chromosomes can disturb signaling pathways that impact the expression of a set of genes performing certain biological functions. Integrating differentially expressed genes and pathways can lead to discovering higher-level disease-associated networks. The Mid-South Bioinformatics Centre (MBC) and Joint Bioinformatics Ph.D. Program of University of Arkansas at Little Rock and University of Arkansas for Medical Sciences (UALR-UAMS) are particularly interested in promoting education and research advancement in comprehensive translational bioinformatics studies. To promote synergistic education and research in translational bioinformatics, the International Society of Intelligent Biological Medicine (ISIBM) provided academic sponsorship to the 2014 International Conference on Bioinformatics and Computational Biology. MBC works with UALR-UAMS in promoting cutting-edge translational bioinformatics research.

The 2014 International Conference on Bioinformatics and Computational Biology received large pool of hundreds of paper submissions. All submitted papers were peer reviewed by the conference program committee members (http://www.world-academy-of-science.org/worldcomp14/ws/conferences/biocomp14/committee) and invited external experts. Six papers selected to this special *BMC Bioinformatics *supplement represent current trends in the cutting-edge translational bioinformatics research and were chosen from large submissions by the review committee chaired by ISIBM President Dr. A. Keith Dunker (Founding Director and T. K. Li Professor for Medical Research of Indiana University School of Medicine Centre for Computational Biology and Bioinformatics) based on peer-reviews. ISIBM Vice-President Dr. Hamid R. Arabnia (Editor-in-Chief of Journal of Supercomputing, Professor of Computer Science at University of Georgia), Vice-President Dr. Dong Xu (Editor-in-Chief of International Journal of Functional Informatics and Personalised Medicine, Associate Editor-in-Chief of IEEE/ACM Transactions on Computational Biology and Bioinformatics, James C. Dowell Professor and Chair of Computer Science Department at University of Missouri), Secretary-General Dr. Yunlong Liu (Associate Editor of BMC Genomics, Associate Professor of Molecular and Medical Genetics and Director of Bioinformatics Core at Indiana University School of Medicine, Indiana University Purdue University Indianapolis), Dr. Zhongxue Chen (Associate Editor of BMC Genomics and Director of Study Design and Data Analysis Consulting Centre of Indiana University at Bloomington), Dr. Xiang Qin (Assistant Professor of Molecular and Human Genetics at the Human Genome Sequencing Centre at Baylor College of Medicine), along with Dr. Weida Tong (HHS/FDA/NCTR Director of Bioinformatics and Biostatistics and Professor of Bioinformatics by courtesy at University of Arkansas at Little Rock, and core faculty of joint bioinformatics Ph.D. program), and Dr. Youping Deng (Associate Editor of BMC Research Notes, Associate Professor of Medicine and Director of Cancer Bioinformatics and Biostatistics of Rush University Medical Centre in Chicago) also served on the review committee. External experts were invited to review the submitted papers and the committee finally select these six significant papers [1-6] for the BMC Bioinformatics supplement based on peer-reviews.

In this *BMC Bioinformatics *supplement, Yang and Elnitski's laboratories performed a series of in-depth investigations and identified bidirectional promoters and conservations of this type of promoters utilizing orthologous mapping in human and mouse genomes [1]. They incorporated data generated by cap analysis gene expression (CAGE), and validated most of 5' end of UCSC Genome Browser annotations that were used in the study. Their studies of coordinated expression of bidirectional gene pairs were significant as the conserved bidirectional promoters in humans have been implicated in complex diseases such as cancer. While mouse has been used as a standard model animal for investigating a variety of diseases in humans, compassion with human genes revealed that bidirectional promoters regulate significant amounts of genes in mammalian genomes, especially regulate disease associated genes. Understanding mechanisms of these promoters based on their functional roles and evolutional patterns can provide valuable resources to further understanding of gene regulation, transcriptional mechanisms and their roles in the disease transformation. In addition, Yang and Elnitski's laboratories found that the gene expression mediated through bidirectional promoters can influence many biological processes such as histone modification. Furthermore, correlations between bidirectional promoters and lncRNAs (long non-coding RNAs) identified by Yang are considered as highly significant because such findings can facilitate the identification of functions of lncRNAs in connection with the regulatory roles of bidirectional promoters. This important work offers deeper insights into the regulatory roles of bidirectional promoters in connection with lncRNAs in complex diseases.

Equally impressive is the work of Yang's laboratory to systematically investigate TCGA (The Cancer Genome Atlas) real cancer data. The advent of high-throughput next-generation sequencing technologies marked the beginning of a new era for personalized medicine research. The impact of having an individual genome and personalized genomic data in hands generates high demands of developing more powerful computational approaches to handle massive information imbedded in the big data, which will obviously generate profound effect on how data-intense biomedical research shall be conducted toward the improvement of human health and lifesavings. Yang's laboratory utilized TCGA RNA-Seq data from more than 500 kidney renal clear cell carcinoma (KIRC) patients to investigate genes and pathways that were significantly altered in the disease [2]. The laboratory identified 186 genes with significant differential expressions between normal and disease samples. They found four-subtypes of the kidney cancer, which were consistent with the results of recent publications. In addition, an intelligent SVM (support vector machine) based supervised classifier was built using the identified differentially expressed genes to predict unknown samples. The intelligent machine can effectively distinguish cancer samples from non-cancer samples with high accuracy. By integrating differentially expressed genes with pathway analysis, the authors revealed several putative pathways disrupted in the disease. Their results not only confirmed a number of previously reported disease pathways in literature, but also identified new roles of pathways in the disease that has not been well studied yet. Furthermore, based on the differentially expressed genes between tumor and normal tissue samples, results from their network analysis suggested that combining differentially expressed genes, pathways and networks can infer the upstream regulators, which can ultimately help identifying disease causal genomic mutations. The research was a part of the plenary invited talk entitled "Integrative systems biology approaches to identify disrupted pathways in disease development" (http://www.world-academy-of-science.org/worldcomp14/ws/keynotes/invited_talk_yang ). The integrative methods presented from Yang's laboratory demonstrated that combining differentially expressed genes, gene networks and biological pathways have provided powerful approaches to further reveal underlying disease mechanisms and effective drug targets.

Effectively combining information from individual studies is critical to assess rare variants in GWAS (genome-wide association study). Chen's laboratory and collaborators developed a novel statistical approach using the inverse of the p-value as the shape parameter in the gamma distribution to more effectively combing p-values from individual studies [3]. This approach can adaptively choose the shape parameter of the gamma distribution for each individual study, since the flexibility in choosing the parameters allows effectively combing p-values for either homogenous or heterogeneous individual studies. This is considered as an improved method to assess the genomic variants association with diseases, especially to handle the heterogeneous cases in complex diseases. Chen's laboratory and collaborators demonstrated that the performance of their new method outperformed existing approaches when the effects among the studies are more heterogeneous. The newly developed approach has been tested favourably in genome-wide association study.

Intrinsically disordered proteins (IDP) play important roles in many biological processes that include post-translational modifications, entropic chain spring-based restoring forces, flexible linkers, signal transduction, protein aggregation and coupled folding and binding. Dunker's laboratory developed new the IDP-Hydropathy scale using the C-H (charge-hydropathy) plot as the classifier based on sets of sequences that fold into 3D structure as compared to collections of sequences that do not fold [4]. The method provided a measurement of how various amino acids contribute to protein folding using the property of hydropathy. For many years, Dunker's laboratory has taken a lead in classifying proteins that either fold into 3D structures or do not fold into any structure intrinsically. The paper certainly aided to the advancement of this very important but not widely known field. Dunker's laboratory provided a new approach to accurately classify structured and disordered proteins based on hydropathy using the C-H plot. The authors reported 19 different hydropathy scales including Kyte & Doolittle scale. They compared the predicting accuracy of the C-H plot method using different hydropathy scales. They used support vector machine (SVM) to train the classifier that discriminates structured proteins from disordered proteins. The weights produced by the SVM are then used as a new hydropathy scale for the C-H plot. Their new hydropathy scale was used to boost the predictive power of the classifier. They concluded that their IDP-Hydropathy would likely be the best scale to use for any type of algorithm developed to predict protein disorder.

Lu and Deng's laboratories and collaborators performed differentially expressed gene analysis using microarray and genome-wide expression profile of Type II diabetes (T2D) [5]. Using blood samples from healthy humans, pre-diabetic and diabetes patients, they identified 79 differentially expressed genes with fold change larger than 2. They built a discriminant model using expression levels of 79 differential genes in combination with clinical factors that include age, sex, and race to achieve over 91% accuracy in diagnosing / predicting status of the T2D (normal people, pre-diabetic patients and T2D patients). Their Gene Ontology (GO) analysis revealed a collection of significant GO term associated with the differential genes. Their work can provide a combined molecular and pedigree analytic method that could potentially lead to an effective screening tool for identifying overall health or illness of humans and predicting progression of the disease development. The pairwise analysis presented in the paper is significant and innovative. Combining gene expression, pathway and network analysis can reveal underlying molecular mechanisms for better preventing, diagnosing and treating the disease.

United States Foods and Drug Administration (FDA) has been the leader in drug toxicity studies, Tong's laboratory at FDA assessed safety of drugs and the impact on human health from drug toxicity [6]. Medical drugs are not natural products and often cause damages to livers and human health. Unfortunately, toxicity of many drugs has not been fully studied. The investigators from United States Foods and Drug Administration and University of Arkansas at Little Rock fully understand the importance of a comprehensive assessment of drug safety and toxicity. They developed a method to systematically search the literature and gathered information together to present the risk of accurate liver failure, which can be a fatal consequence of certain drugs. This research opens a new opportunity to comprehensively identify potential outcomes of certain drugs using acute liver failure as case studies. Results from the research can be potentially useful in future personalized genomics and individualized healthcare investigations.

## Conclusion

Integrating multi-layer genomic data has helped to reveal many underlying molecular mechanisms. In particular, identifying roles of bidirectional promoters with cancer-related genes using different genome-scale data can systematically assess genomic mutations and gene expression that are associated with dysfunctional regulations in cells and/or malignant transformation, while combining gene expression and pathway analysis with gene networks using systems biology approaches can help revealing underlying disease mechanisms and link pathways to disrupted gene networks in disease development. The cutting-edge research presented in this BMC Bioinformatics special supplement represents the current development of computational approaches in different bioinformatics studies. Developing approaches to combine the information from different data helps the advances in translational bioinformatics, which ultimately facilitate revealing the landscape of complex disease mechanisms.

## Competing interests

The authors declare that they have no competing interests.

## Authors' contributions

AKD, YD, HRA, XQ, LW, ZC, ZL, YL, AN, JSL, JYY, WT, and DX organized external peer-reviews, contributed to reviews and participated in the selection of five high-quality research papers for the special BMC Genomics supplement based on peer-reviews. MQY and WY wrote the article which was read and approved by all authors.

## Declaration

The funding for publication of the article has come from the MidSouth Bioinformatics Centre, and the Joint Bioinformatics Ph.D. Program of University of Arkansas at Little Rock and University of Arkansas for Medical Sciences with NIH/NIGMS 5P20GM10342913 and ASTA award # 15-B-23.

This article has been published as part of *BMC Bioinformatics *Volume 15 Supplement 17, 2014: Selected articles from the 2014 International Conference on Bioinformatics and Computational Biology: bioinformatics. The full contents of the supplement are available online at http://www.biomedcentral.com/bmcbioinformatics/supplements/15/S17.
